# Healing enhancement of chronic venous stasis ulcers utilizing H-WAVE^® ^device therapy: a case series

**DOI:** 10.1186/1757-1626-3-54

**Published:** 2010-02-10

**Authors:** Kenneth Blum, Amanda LH Chen, Thomas JH Chen, B William Downs, Eric R Braverman, Mallory Kerner, Stella Savarimuthu, Anish Bajaj, Margaret Madigan, Seth H Blum, Gary Reinl, John Giordano, Nicholas DiNubile

**Affiliations:** 1 Department of Psychiatry, University of Florida College of Medicine, Gainesville, Fl, USA; 2 Engineering & Management of Advanced Technology, Chang Jung University, Taiwan, Republic of China; 3 Department of Occupation Health and Safety, Chang Jung University, Taiwan, Republic of China; 4 Department of Neurosurgery, Weill Cornel School of Medicine, New York, NY, USA; 5 Department of Clinical Research, Path Research Foundation, New York, NY, USA; 6 Department of Personalized Medicine, Synaptamine, Inc. San Antonio, Texas, USA; 7 Nautilus, Inc Vancouver, WA, USA; 8 Department of Holistic Medicine, G&G Holistic Addiction Treatment Center (Pain Track) North Miami Beach, Florida, USA; 9 Department of Orthopedic Surgery, Hospital of the University of Pennsylvania, Philadelphia, PA, USA

## Abstract

**Introduction:**

Approximately 15% (more than 2 million individuals, based on these estimates) of all people with diabetes will develop a lower-extremity ulcer during the course of the disease. Ultimately, between 14% and 20% of patients with lower-extremity diabetic ulcers will require amputation of the affected limb. Analysis of the 1995 Medicare claims revealed that lower-extremity ulcer care accounted for $1.45 billion in Medicare costs. Therapies that promote rapid and complete healing and reduce the need for expensive surgical procedures would impact these costs substantially. One such example is the electrotherapeutic modality utilizing the H-Wave^® ^device therapy and program.

It has been recently shown in acute animal experiments that the H-Wave® device stimulation induces a nitric oxide-dependent increase in microcirculation of the rat Cremaster skeletal muscle. Moreover, chronic H-wave® device stimulation of rat hind limbs not only increases blood flow but induces measured angiogenesis. Coupling these findings strongly suggests that H-Wave® device stimulation promotes rapid and complete healing without need of expensive surgical procedures.

**Case presentation:**

We decided to do a preliminary evaluation of the H-Wave^® ^device therapy and program in three seriously afflicted diabetic patients. Patient 1 had chronic venous stasis for 6 years. Patient 2 had chronic recurrent leg ulcerations. Patient 3 had a chronic venous stasis ulcer for 2 years. All were dispensed a home H-Wave^® ^unit. Patient 1 had no other treatment, patient 2 had H-Wave^® ^therapy along with traditional compressive therapy, and patient 3 had no other therapy.

For patient 1, following treatment the ulcer completely healed with the H-Wave^® ^device and program after 3 months. For patient 2, by one month complete ulcer closure occurred. Patient 3 had a completely healed ulcer after 9 months.

**Conclusions:**

While most diabetic ulcers can be treated successfully on an outpatient basis, a significant proportion will persist and become infected. Based on this preliminary case series investigation we found that three patients prescribed H-Wave^® ^home treatment demonstrate accelerated healing with excellent results. While these results are encouraging, additional large scale investigation is warranted before any interpretation is given to these interesting outcomes.

## Introduction

The worldwide increase in prevalence of type 2 diabetes has resulted in a parallel increase in diabetic foot ulcers, which is a pervasive and significant problem associated with this disease [1]. Currently, an estimated 10.3 million people have been diagnosed with diabetes, while an additional estimated 5.4 million people with diabetes remain undiagnosed, representing a six-fold increase in the incidence of diabetes over the past four decades [2]. Approximately 15% (more than 2 million individuals, based on these estimates) of all people with diabetes will develop a lower-extremity ulcer during the course of the disease [3]. While most of these ulcers can be treated successfully on an outpatient basis, some will persist and become infected. Ultimately, between 14% and 20% of patients with lower-extremity diabetic ulcers will require amputation of the affected limb [4]. Diabetic foot ulcers can result in staggering financial burdens for both the healthcare system and the patient. For example, analysis of the 1995 Medicare claims revealed that lower-extremity ulcer care accounted for $1.45 billion in Medicare costs and contributed substantially to the high cost of care for diabetics, compared with Medicare costs for the general population [5]. A search in PUBMED revealed that there has not been an update published on the actual cost of Medicare for diabetic ulcers, however the cost as stated earlier they have been increasing at the rate of six-fold over the last four decades [6]. While there are other conditions that result in chronic venous stasis ulcers such as vein striping failed surgery, diabetes is a major etiology of this condition. Therapies that promote rapid and complete healing and reduce the need for expensive surgical procedures would impact these costs substantially.

It is important to note that while the etiology of diabetic foot ulcers is the impairment of microcirculation and autonomic dysfunction, the causes of chronic venous insufficiency are multiple. One of the possible causes of chronic venous insufficiency is extraluminal lipoma with common femoral vein obstruction. However it is also of note that the etiology may be best explained by the well-known valve cusp hypothesis. In this scenario firstly, should the foregoing events not proceed to frank thrombogenesis, the valves may nevertheless be chronically injured and become incompetent. Serial incompetence in lower limb valves may then generate "passive" venous hypertension. Secondly, should ostial valve thrombosis obstruct venous return from muscles via tributaries draining into the femoral vein, "active" venous hypertension may supervene. Muscle contraction would force the blood in the vessels behind the blocked ostial valves to re-route. Passive or active venous hypertension opposes return flow, leading to luminal hypoxemia and vein wall distension, which in turn may impair vasa venarum perfusion; the resulting mural endothelial hypoxia would lead to leukocyte invasion of the wall and remodeling of the media.

It has been recently shown in acute animal experiments that the H-wave® (Electronic WaveForm Lab, Huntington, Beach, California) device stimulation (HWDS) induces a nitric oxide (NO)-dependent increase in microcirculation of the rat Cremaster skeletal muscle [7]. Moreover, chronic HWDS of rat hind limbs not only significantly increases blood flow (above 247% from baseline) but induces measured angiogenesis [7]. Coupling these findings strongly suggests that HWDS promotes rapid and complete healing without the need of expensive surgical procedures. With this in mind, we decided to preliminary evaluate H-Wave^® ^device therapy and program in three seriously inflicted diabetic patients with chronic venous stasis ulcers.

## Case presentation

### Methods

In this preliminary case series we selected three seriously inflicted patients with chronic venous stasis ulcers. Each patient signed an informed consent. The study was evaluated and approved by the Path Research Foundation IRB committee (NIH registration #IRB0002334). The study was designed and executed by one of us (MSA) and subsequently approved by the other authors. The setting for this case study was LACUSC Medical Center. In each case the location and size in cm were denoted pre and post H-Wave^® ^treatment. At initial contact each patient was dispensed a home H-Wave^® ^device and provided follow-up instruction by a representative. There were three different scenarios executed in this study: 1) H-Wave^® ^therapy alone; 2) H-wave® therapy with traditional compression therapy; 3) H-Wave^® ^therapy and weekly wound care.

### H-Wave^® ^Therapy Regimen

Patient one received a two-channel home H-wave® device at the beginning of the study period and used it throughout. Patient two received only once weekly treatments with the three channel clinical H-wave® device. Patient three started with once weekly clinical H-wave® treatments, but after nine months received a home H-wave® device to use daily in addition to once weekly treatments.

Home treatment was given with a two channel portable H-wave® device. The patient was instructed to self treat for at least one hour per day. The pads from the first channel were placed on the quadriceps muscle of the affected leg. The pads from the second channel were placed on the gastrocnemius and over the head of the fibula. The frequency dial of the H-wave® device was set to minimum (1-2 Hz) to create rhythmic non-fatiguing muscle contractions. The intensity dial was increased to at or near maximum to create strong muscle contractions.

In clinic treatment was given with a three channel clinical H-wave® device.

Treatment times were between 30 and 60 minutes. The pads from the first channel were placed on the quadriceps muscle of the affected leg. The pads from the second channel were placed on the gastrocnemius and over the head of the fibula. The pads from the third channel were placed on the top and bottom of the foot. The frequency dial of the H-wave® device was set to minimum (1-2 Hz) to create rhythmic non-fatiguing muscle contractions. The intensity dial was increased to at or near maximum to create strong muscle contractions.

Both H-wave® models have identical waveforms and output parameters, the difference is only in the number of output channels and therefore electrodes that can be placed one the skin.

### Wound Care Procedure

Our approach to the utilization of standard wound care was palliative in nature. Thus this approach can be summarized with the mnemonic S-P-E-C-I-AL (S = stabilizing the wound, P = preventing new wounds, E = eliminate odor, C = control pain, I = infection prophylaxis, A = advanced, absorbent wound dressings, L = lessen dressing changes) as described by Alvarez et al. [8]. Our approach to wound healing was based on the National Guideline Clearinghouse report and recommendations. The report included summary algorithm for venous ulcer care with annotations of available evidence [9]. The diagnosis of venous stasis ulcers was confirmed by the following process:

1) **Patient history **prior phlebitis, deep vein thrombosis, lower leg swelling/edema, ache or tiredness in leg, trauma/intimal damage, maternal venous ulcer

2) **Differential diagnosis **Plethysmography, elevated temperature

3) **Physical exam **clinical severity, etiology, anatomy, pathophysiology, edema, stasis dermatitis, measure ulcer size. The following standard of care was adopted in the wound care procedure: manage of peri-wound skin, local wound care, maintain moist wound environment for healing or venous ulcer pain management, antimicrobial wound care and dressings. Patient(s) that received compression therapy conformed to standard practice as denoted by Arnold et al. [10].

### Inclusion/exclusion criteria

It is noteworthy that all of these patients were uninsured and were treated free of charge. The three patients were part of an approved IRB larger study. The the three patients were ambulatory out-patients and met all criteria for inclusion of this study.

### Inclusion

One major inclusion criteria into the study was that each patient had to have diabetes for more than 24 months. They had to be ambulatory and not satisfied with any previous treatment. All patients had to have chronic venous stasis ulcers.

### Exclusion

The main purpose of this series was to assess the benefits of administering the H-wave® device^® ^in the treatment of chronic diabetic ulcers. Therefore, we utilized strict inclusion criteria which was approved as part of the larger study. Exclusion consisted of having: serious co-morbid cardiovascular problems, arterial insufficiency, cancer of any type, addiction to any psychoactive drug, or be currently taking medications for existing conditions other than anti-diabetic agents.

None of the patient's underwent angiographic assessment of their lower limb circulation or other techniques such as prostaglandin analogues.

### Results

The following information is provided on each patient including the actual progression of healing of the ulcer. This information was obtained by the staff of clinic at LACUSC Medical Center under the clinical supervision of MSA.

#### Case report 1

**Figure 1:**
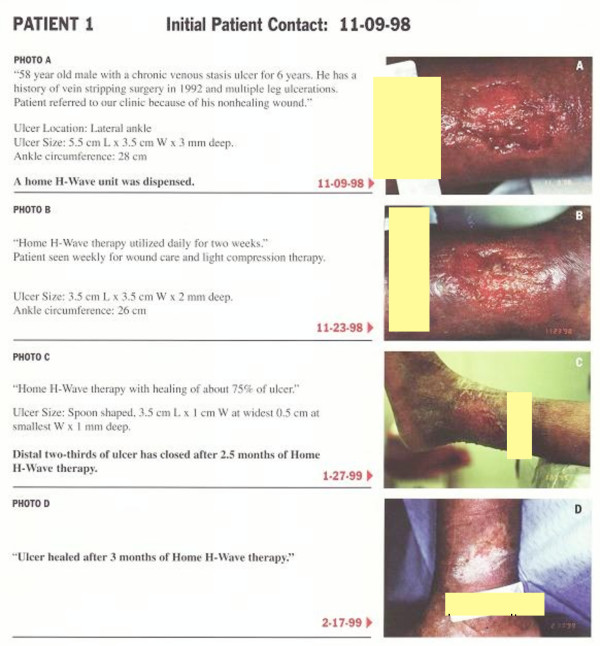
**Cumulative Wound healing pictures of Patient #1**.

The first patient was a 58 year old Caucasian male with a chronic venous stasis ulcer of the lateral ankle. This patient had a history of vein stripping surgery in 1992 and multiple leg ulcerations. The patient was referred to the LACUSC Medical Center Clinic because of his non-healing ulcerated wound. At the initial contact on 11-09-98 the ulcer size measured 5.5 cm L. × 3.5 cm W × 3 mm deep with an ankle circumference of 28 cm (See figure 1 photo A). At this date the patient was dispensed a home H-wave^® ^unit. On 11-23-98, figure 1 photo B shows the improvement following home H-Wave^® ^therapy used daily for two weeks. In addition this patient was seen weekly for wound care and light compression therapy. At this date the ulcer size measured 3.5 cm L × 3.5 cm W × 2 mm deep with an ankle circumference of 26 cm. On 11-27-99, figure 1 photo C shows healing of about 75% or distal two-thirds of ulcer has closed after 2.5 months of home H-Wave^® ^therapy. At this date the ulcer size measured 3.5 cm L × 1 cm W at widest 0.5 cm at smallest W × 1 mm deep and spoon shaped. Finally on 2-17-99, Figure 1 photo D shows complete healing of ankle ulcer after 3 months of H-Wave^® ^therapy.

#### Case report 2

**Figure 2:**
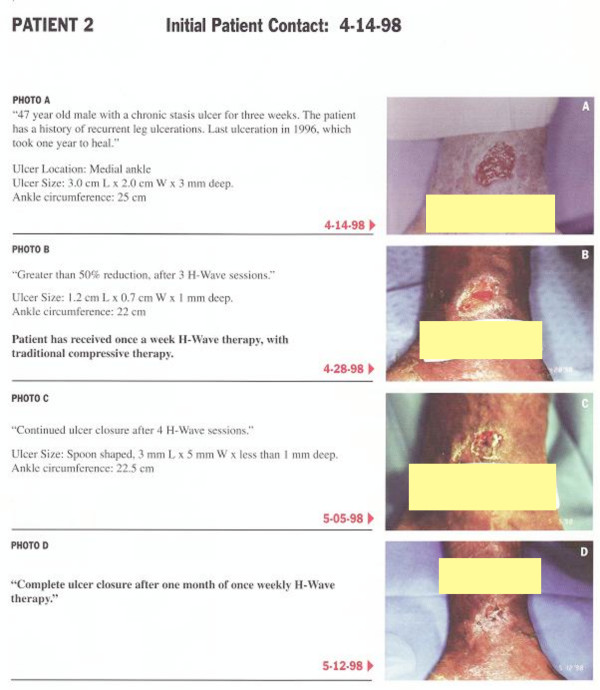
**Cumulative Wound healing pictures of Patient # 2**.

The second patient was a 47 year old African-American male with a chronic venous stasis ulcer of the medial ankle. This patient had a history of recurrent leg ulcerations. The last ulceration in 1996 took one year to heal. The patient was referred to the LACUSC Medical Center Clinic because of his non-healing ulcerated wound. At the initial contact on 4-14-98 the ulcer size measured 3.0 cm L. × 2.0 cm W × 3 mm deep with an ankle circumference of 25 cm (See figure 2 photo A). On 4-28-98, figure 2 photo B shows the improvement of greater than 50% following H-Wave^® ^therapy after 3 H-wave® sessions. The patient received once a week H-wave® therapy at the clinic. In addition this patient received traditional compressive therapy. At this date the ulcer size measured 1.2 cm L × 0.7 cm W × 1 mm deep with an ankle circumference of 22 cm. On 5-05-98, figure 1 photo C shows continued closure after 4 H-wave® sessions. At this date the ulcer size measured 3.0 mm L × 5 mm W × less than 1 mm with an ankle circumference of 22.5 cm. and spoon shaped. Finally on 5-12-98, Figure 1 photo D shows complete healing of ankle ulcer after one month of once weekly of H-Wave^® ^therapy.

#### Case report 3

**Figure 3:**
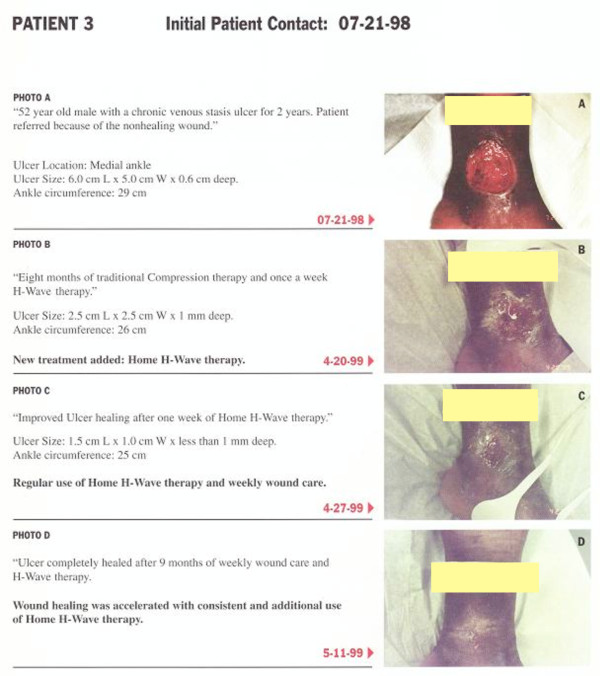
**Cumulative Wound healing pictures of Patient # 3**.

The third patient was a 52 year old African American male with a chronic venous stasis ulcer of the medial ankle for 2 years. The patient was referred to the LACUSC Medical Center Clinic because of his non-healing initial contact on 07-21-98 the ulcer size measured 6.0 cm L. × 5.0 Cm W × 0.6 mm deep with an ankle circumference of 29 cm (See figure 3 photo A). At this date the patient was dispensed a home H-wave^® ^unit. On 04-20-99, figure 3 photo B shows the improvement following home H-Wave^® ^therapy utilized once a week in the clinic. In addition this patient was seen weekly for traditional compression therapy. At this date of eight months of traditional compression therapy and once a week therapy the ulcer size measured 2.5 cm L × 2.5 cm W × 1 mm deep with an ankle circumference of 26 cm. Moreover at this date home H-Wave^® ^therapy was added including weekly wound care. On 04-27-99, figure 3, photo C shows healing improvement after one week of home H-Wave^® ^therapy. At this date the ulcer size measured 1.5 cm L × 1 cm W × less than 1 mm deep with a circumference of 25 cm. Finally on 5-11-99, Figure 3 photo D shows complete healing of ankle ulcer after 9 months of H-Wave^® ^therapy and weekly wound care.

## Discussion

This is the first study that has investigated the effects of HWDS in wound healing and in particular venous stasis ulcers. It is our contention the effects observed in this case series is not surprising in light of the mechanism by which HWDS increases microcirculation in rat experiments [7]. Moreover, nitric oxide is profoundly involved with wound healing by virtue of its effects to control blood flow to tissues and anti-inflammatory effects to influence pain [11]. Interestingly, wound healing represents a particularly challenging clinical problem to which no efficacious treatment regimens currently exist. Although protein-type mediators are well established players in this process, emerging evidence from both animal and human studies indicates that nitric oxide plays a key role in wound repair. The beneficial effects of nitric oxide on wound repair may be attributed to its functional influences on angiogenesis, inflammation, cell proliferation, matrix deposition, and remodeling [12,13]. The H-wave® nitric oxide rat study revealed that the increase in microcirculation induced by H-wave® was blocked by the nitric oxide receptor inhibitor L-NAME (N-monomethylarginine), indicating a nitric oxide-dependent response. It is well established that angiogenesis plays an important role during adult life span, and it is primarily involved in tissue repair mechanisms [14]. Angiogenesis heals injured or fractured body parts by activating genes which ultimately leads to the production of the angiogenic factors such as Vascular Endotherial Growth Factor [VEGF] [15].

Understanding physiological processes involved in wound healing along with the findings related to both nitric oxide dependent increases in microcirculation, as well as significant induction of angiogenesis following chronic HWDS in rats provides a clear mechanism for the H-wave® positive effects.

## Conclusion

Approximately 15% (more than 2 million individuals) of all people with diabetes will develop a lower-extremity ulcer during the course of the disease. While most of these ulcers can be treated successfully on an outpatient basis, a significant proportion will persist and become infected. Based on this preliminary case series investigation we found that three patients prescribed H-Wave^® ^home treatment demonstrate accelerated healing of chronic venous stasis of ankle ulcers with excellent results. While these results are encouraging, additional large scale investigation is warranted before any interpretation is given to these interesting outcomes.

## Consent

Written informed consent was obtained from the patients for publication of this case report and accompanying images. A copy of the written consent is available for review by the Editor-in-Chief of this journal.

## Competing interests

While KB, GR and ND are paid consultants of Electronic Waveform Labs, Huntington Beach, California, they do not have any ownership. There is no other conflict of interest related to this data.

## Authors' contributions

KB developed the writing of the manuscript and directed the publication submission; ALCC contributed to the overall writing of the manuscript and provided comments; TJHC contributed to the literature background and edited the manuscript; BWD contributed to the overall edits of the manuscript and provided important concepts; ERB was responsible for the IRB approval and clinical direction; MK contributed to the overall submission of the manuscript and provided important editorial feedback and development of journal formatting; SS provided important feedback and developed the informed consent forms; AB contributed to editorial review of the final manuscript; MM provided important information on wound care; SHB provided literature search and reference checking including editorial comments; GR provided important conceptual physiological directed comments and literature search; JG provided editorial review of the final manuscript; ND provided important editorial and conceptual comments and consulted on appropriate wound care parameters. All authors read and approved the final manuscript.
